# Development of a computational fluid dynamic model to investigate the hemodynamic impact of REBOA

**DOI:** 10.3389/fphys.2022.1005073

**Published:** 2022-10-13

**Authors:** Antonio C. Renaldo, Magan R. Lane, Sophie R. Shapiro, Fahim Mobin, James E. Jordan, Timothy K. Williams, Lucas P. Neff, F. Scott Gayzik, Elaheh Rahbar

**Affiliations:** ^1^ Department of Biomedical Engineering, Wake Forest School of Medicine, Winston Salem, NC, United States; ^2^ Virginia Tech—Wake Forest University School of Biomedical Engineering and Sciences, Blacksburg, VA, United States; ^3^ Department of Vascular and Endovascular Surgery, Wake Forest School of Medicine, Winston Salem, NC, United States; ^4^ Department of Cardiothoracic Surgery, Wake Forest School of Medicine, Winston Salem, NC, United States; ^5^ Department of General Surgery, Section of Pediatric Surgery, Wake Forest School of Medicine, Winston Salem, NC, United States; ^6^ Center for Injury Biomechanics, Wake Forest School of Medicine, Winston Salem, NC, United States

**Keywords:** REBOA, CFD—computational fluid dynamics modeling, shear rate, shear stress, pressure, blood flow, hemorrhage

## Abstract

**Background:** Resuscitative endovascular balloon occlusion of the aorta (REBOA) is a lifesaving intervention for major truncal hemorrhage. Balloon-tipped arterial catheters are inserted *via* the femoral artery to create a temporary occlusion of the aorta, which minimizes the rate of internal bleeding until definitive surgery can be conducted. There is growing concern over the resultant hypoperfusion and potential damage to tissues and organs downstream of REBOA. To better understand the acute hemodynamic changes imposed by REBOA, we developed a three-dimensional computational fluid dynamic (CFD) model under normal, hemorrhage, and aortic occlusion conditions. The goal was to characterize the acute hemodynamic changes and identify regions within the aortic vascular tree susceptible to abnormal flow and shear stress.

**Methods:** Hemodynamic data from established porcine hemorrhage models were used to build a CFD model. Swine underwent 20% controlled hemorrhage and were randomized to receive a full or partial aortic occlusion. Using CT scans, we generated a pig-specific aortic geometry and imposed physiologically relevant inlet flow and outlet pressure boundary conditions to match *in vivo* data. By assuming non-Newtonian fluid properties, pressure, velocity, and shear stresses were quantified over a cardiac cycle.

**Results:** We observed a significant rise in blood pressure (∼147 mmHg) proximal to REBOA, which resulted in increased flow and shear stress within the ascending aorta. Specifically, we observed high levels of shear stress within the subclavian arteries (22.75 Pa). Alternatively, at the site of full REBOA, wall shear stress was low (0.04 ± 9.07E-4 Pa), but flow oscillations were high (oscillatory shear index of 0.31). Comparatively, partial REBOA elevated shear levels to 84.14 ± 19.50 Pa and reduced flow oscillations. Our numerical simulations were congruent within 5% of averaged porcine experimental data over a cardiac cycle.

**Conclusion:** This CFD model is the first to our knowledge to quantify the acute hemodynamic changes imposed by REBOA. We identified areas of low shear stress near the site of occlusion and high shear stress in the subclavian arteries. Future studies are needed to determine the optimal design parameters of endovascular hemorrhage control devices that can minimize flow perturbations and areas of high shear.

## Introduction

Failure to control hemorrhage is the leading cause of potentially preventable death in trauma and accounts for 91% of military and 35% of civilian fatalities (Trunkey and Lim, 1974; Eastridge et al., 2019). Non-compressible torso hemorrhage, referring to regions of the body that cannot be controlled with a tourniquet (e.g., abdomen, thoracic cavity), pose a serious threat to one’s survival. Nearly 34% of non-compressible torso hemorrhage fatalities occur prior to hospital admission warranting the need for quick hemorrhage control (Sauaia et al., 1995). To address this problem, minimally invasive endovascular hemorrhage control (EHC) devices have been proposed as a more efficient and less invasive way to minimize blood loss and prolong survival.

**FIGURE 1:**
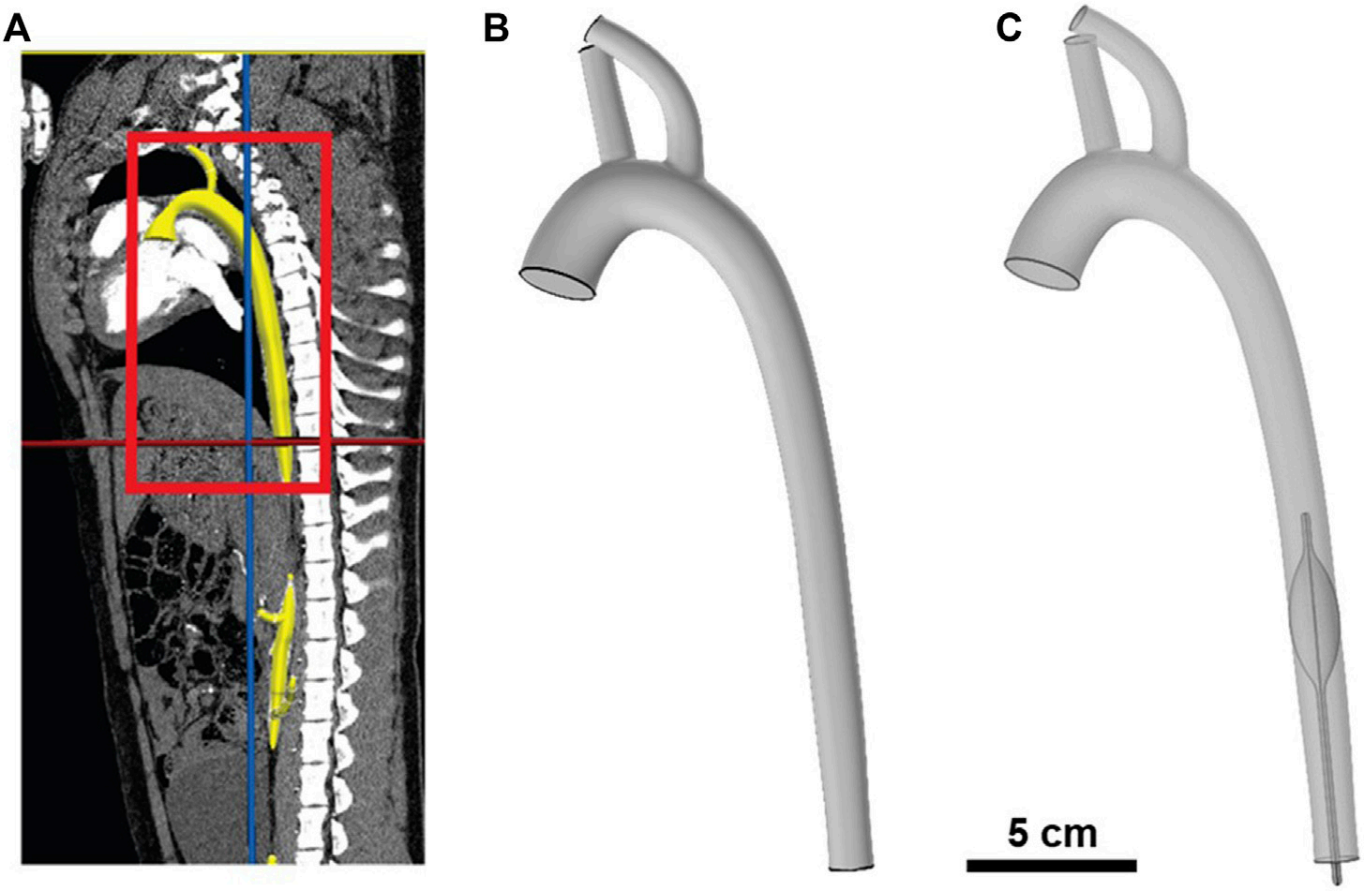
Illustration of porcine aorta computational model. **(A)** Segmentation of porcine aorta from computed tomography (CT) scan; aorta is highlighted in yellow and the region of interest is boxed in red. **(B)** Geometric 3D model of the porcine aorta. **(C)** Geometric 3D model of REBOA placement in Zone I (i.e., proximal to diaphragm).

Resuscitative Endovascular Balloon of the Aorta (REBOA) is the most popular EHC device; it has become increasingly adopted as a minimally invasive clinical intervention for the treatment of non-compressible hemorrhage. REBOA involves the temporary inflation of a balloon catheter in the aorta, which restricts blood flow distal to the balloon, and consequently minimizes bleeding. While this technique is effective in restoring proximal perfusion, it can only be applied for short periods of time before deleterious downstream effects of the aortic occlusion (i.e., ischemia, organ failure, etc.) start to outweigh its initial benefit (Stannard et al., 2011; Williams et al., 2018; King, 2019). At present, one of the greatest limitations to REBOA is the ischemia-reperfusion (I/R) injury and subsequent kidney injury/renal failure that manifests after imposing a full aortic occlusion. There have even been some reports of endothelial damage either directly from the balloon catheter or subsequent hemodynamic or ischemia alterations after REBOA. However, the direct cause of these endothelial dysfunctions and acute coagulopathy is not well understood (Thrailkill et al., 2021). Pre-clinical large animal studies suggest that Zone 1 REBOA (proximal to diaphragm, Figure 1) is survivable for up to 60 min and Zone 3 (distal to renal artery, Figure 1) for 90 min. In humans, however, Zone 1 REBOA has been consistently lethal at 45 min (Markov et al., 2013; Scott et al., 2013; Norii et al., 2015; Doucet and Coimbra, 2017).

Current research is focused on optimizing the implementation of REBOA and other EHC devices by performing experiments with large animal models of hemorrhagic shock. While swine studies have been important drivers of REBOA research, they are limited in providing a complete quantitative depiction of the acute hemodynamic and physiologic response during full or partial aortic occlusion (Clendenen et al., 2017; Reisz et al., 2018; Williams et al., 2018). Monitoring blood pressure and flow rates at discrete locations within the arterial tree is highly invasive and provides only a snapshot of the hemodynamic response. Arguably, the lack of alternative robust preclinical platforms to investigate the fundamental hemodynamics and physiological mechanisms related to hemorrhage and REBOA has impeded research progress in this field. Additionally, current preclinical animal models are expensive and time and resource intensive.

Computational fluid dynamic (CFD) models offer the opportunity to simulate the acute hemodynamic response in a precise and more cost-effective manner. Such models have been extensively used to study blood flow in a variety of cardiovascular diseases (such as aneurysms and thrombosis), device optimization, and even surgical planning (Rahbar et al., 2011; Stewart et al., 2012; Arthurs et al., 2021). However, no CFD study, to the best of our knowledge, has been conducted to investigate the effects of balloon occlusion within the aorta mimicking REBOA implementation. We postulate that better quantification of the hemodynamics can provide insights into the role biomechanics play in regulating the acute response to partial vs. full REBOA. In this study, our primary goals were to 1) to quantify the flow and shear stresses near and around the occlusion during a Zone 1 REBOA, and 2) compare the acute hemodynamic changes between partial vs. full aortic occlusion after hemorrhage. We developed CFD models simulating blood flow during normal conditions (Baseline), end of hemorrhage (Hemorrhage), and during full (f-REBOA) and partial (p-REBOA) aortic occlusion. The naming convention distinguishing the degree of aortic occlusion varies across literature, as a full aortic occlusion can be referred to as REBOA, complete REBOA (c-REBOA) or full REBOA (f-REBOA) (Russo et al., 2016b; Williams et al., 2017; Singer et al., 2020). For the purpose of this work, we will refer to models simulating a full occlusion as f-REBOA and partial occlusion as p-REBOA. Flow and pressure parameters from swine experiments were leveraged to construct and calibrate the CFD model. Simulated hemodynamic results were compared to the experimental hemodynamic data to ensure congruent findings and to minimize the bounds of error from the numerical simulations. Key hemodynamic parameters of interest were pressure, velocity, shear stress and oscillatory shear index. All simulations were successfully calibrated to match *in vivo* data. The CFD models developed in this study provide a first glance into the high shear/high flow environment around the balloon occlusion when high degree partial occlusions were imposed.

## Materials and methods

### Animal model of hemorrhage and REBOA

All animal experiments were performed in accordance with the Guide for Care and Use of Laboratory Animals and under protocols approved by the Wake Forest Institutional Animal Care and Use Committee (IACUC). Yorkshire swine (Oak Hill Genetics, Ewing Illinois) underwent a controlled hemorrhage of 20% blood volume for 30 min followed by 20 min of Zone 1 f-REBOA (*n* = 4) or p-REBOA (*n* = 6). Before experimentation, swine were instrumented with a flow probe in the descending aorta, proximal to site of balloon occlusion and pressure probes at the lower left subclavian and femoral arteries as previously described (Tibbits et al., 2018; Williams et al., 2018). These hemodynamic parameters were continuously recorded in PowerLab (ADInstruments, Colorado Springs, CO, United States). Additionally, a Swan Ganz catheter was placed in the pulmonary artery to obtain cardiac output (CO), and continuously recorded in Vital Recorder (Lee and Jung, 2018). Animals were euthanized at the end of experimentation.

### Computed tomography imaging

Contrast-enhanced computed tomography (CT) scanning (GE 64-slice PET/CT Discovery MI DR Scanner) was performed on a 60 kg Yorkshire female swine, placed in a supine position (Oak Hill Genetics, Ewing Illinois), and under general anesthesia. The pig was anesthetized with Telazol and then maintained with 1%–4% isoflurane during imaging. A CT scan based on our institutional protocol (i.e., Wake Forest’s Translational Imaging Program) was conducted to obtain high-resolution images of the aorta and its main arterial branches. The scanning range was the head to the femoral plane. For each scan, a 100 ml injection of Omnipaque contrast (GE Healthcare) was used. The animal was euthanized humanely at the end of imaging.

### Image reconstruction for three-dimensional vascular geometry

The CT scans were imported and segmented in the open-source computational hemodynamics platform, CRIMSON^®^ (v.1.4.5, Ann Arbor MI) (Arthurs et al., 2021) for 3-D reconstruction. The CRIMSON GUI (Graphical User Interface) was used to create a 3D vascular model of the pig aorta with its main branches, which included the brachiocephalic trunk and left subclavian artery, which we refer to as the supra-aortic arteries. The reconstruction areas ranged from the aortic root to the descending aorta. Centerline paths were determined for each artery of interest and circular contours were added perpendicular to the centerline with discrete spacing to represent the vascular lumen. Through the use of lofting functions between contours and applying a union function to blend the aorta and main branching vessels, we obtained a pig-specific 3D geometry. For this CFD study, arterial branches below the balloon (e.g., superior mesenteric, celiac, renal, inferior mesenteric, and iliac) were excluded due to limitations in pressure measurements at these locations. Our primary goal for this initial study was to quantify the acute changes in flow and shear stress near and around the occlusion in both full and partial REBOA when placed in Zone 1. The 3D model was saved in a stereolithographic (STL) format then imported into Geomagic Studio^®^ (Foundation 2014, NC) for modifications and smoothing. The geometry was discretized using a tetrahedral mesh in COMSOL to render one inlet corresponding to the ascending aorta and three outlets corresponding to the two supra-aortic arteries and descending aorta, as seen in Figure 1.

### Computational methods

The hemodynamic simulations were performed using a commercially available computational CFD software package (COMSOL Multiphysics, version 5.6, Burlington MA, United States). The built-in COMSOL meshing tool was employed to discretize the pig-specific vascular geometry, with and without REBOA placement in Zone 1 (i.e., proximal to diaphragm), using tetrahedral grid elements. In the sections below, we provide details about each step of the computational model development.

### 3D REBOA balloon geometry

A representative balloon catheter geometry (10.5 cm in length and max. diameter of 1.38 cm) of an ER-REBOA™ (Prytime Medical Devices, Inc., Boerne, TX, United States) was created within COMSOL. A boolean operation was then used to subtract the geometry from the fluid domain, mimicking an aortic obstruction observed with a high gradient occlusion. To mimic a full aortic occlusion in the f-REBOA model, a cut plane was placed at the center of the balloon geometry without a prescribed outlet boundary condition, thereby creating a wall. In the p-REBOA model, this cut plane was removed, allowing flow past the occlusion.

### Boundary conditions

**FIGURE 2:**
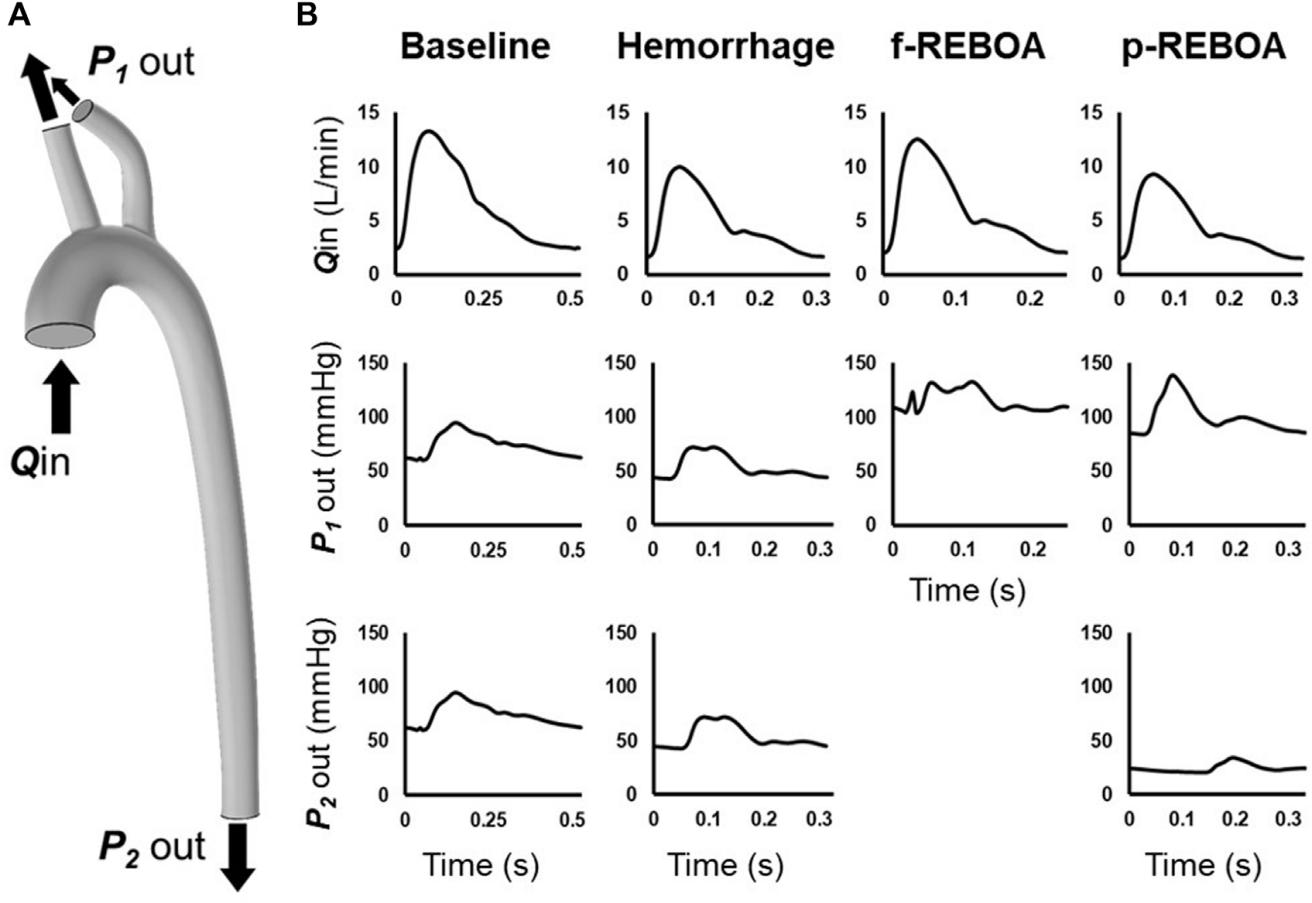
Computational model boundary conditions. **(A)** Location of imposed boundary conditions on 3-D aortic geometry. A flow inlet was prescribed at the ascending aorta (*Q*_in_). Pressure outlets were prescribed to supra-aortic vessels (*P*_1_ out) and descending thoracic aorta (*P*_2_ out) **(B)** Inflow and outlet pressure waveforms for each model. Flow rate at the ascending aorta Q_in_ was estimated by scaling the *in vivo* distal flow waveform from the distal flow probe in the descending aorta to *in vivo* data output *via* a Swan Ganz catheter. Supra-aortic pressure outlets, P_1_ out, were measured at the left subclavian artery and coupled. P_2_ out was iteratively defined to calibrate our model to the available *in vivo* data.

We aimed to simulate the hemodynamics during three key physiologic states: Baseline (normal conditions), End of Hemorrhage (30 min after a controlled hemorrhage of 20% blood loss), and Zone 1 aortic occlusion (15 min after REBOA implementation). A simulated ascending arterial blood flow rate waveform was applied to the inlet and pressure waveforms were set at each outlet (Figure 2A). Swan-Ganz catheter and proximal pressure probe data from the animal experiments were used to approximate blood flow at the inlet and pressure at the supra-aortic vessel outlets (Figure 2B). A summary of CO, heart rate (HR) and mean arterial pressure (MAP) values from the porcine experiments has been provided in Table 1. We assumed that pressure in the two supra-aortic outlets were similar, and thus prescribed the same pressure outlet value for each of these arteries. The pressure outlet in the thoracic aorta was iteratively prescribed to match the experimental data. Figure 2 illustrates the inlet waveforms, associated values of CO and pressure outlet waveforms for each simulation phase. In summary, we simulated an average CO and heart rate of 6.5 L/min and 114 bpm in the Baseline model, 4.8 L/min and 192 bpm in the Hemorrhage model, 6 L/min and 240 bpm in the f-REBOA model and 4.5 L/min and 180 bpm in the p-REBOA model.

**TABLE 1:** Porcine hemodynamic characteristics in the f-REBOA and p-REBOA groups.

	f-REBOA (*n* = 4)			p-REBOA (*n* = 6)		
	T0	T30	T60	T0	T30	T60
	Baseline	Hemorrhage	Occlusion	Baseline	Hemorrhage	Occlusion
CO (L/min)	6.9 ± 0.9	4.7 ± 0.7	6.9 ± 0.9	6.1 ± 1.2	3.8 ± 0.8	4.6 ± 1.4
HR (bpm)	126 ± 22	176 ± 7	207 ± 17	128 ± 22	177 ± 23	171 ± 33
MAP (mmHg)	83 ± 7	63 ± 7	136 ± 17	82 ± 5	55 ± 7	114 ± 9

These experimental hemodynamic parameters were used to generate a corresponding flow inlet waveform for baseline, hemorrhage, f-REBOA, and p-REBOA simulations.

### Non-Newtonian fluid

Blood was modelled as an incompressible, homogeneous, Non-Newtonian fluid. Although blood can be acceptably modelled as Newtonian in large vessels, we chose a Non-Newtonian model to obtain more reliable wall shear stress estimates. A Carreau-Yasuda model was used to simulate the behavior of blood, with a density of 1,060 kg/m^3^. Blood viscosity (*µ*) was defined was:

$$\mu =\mu_{\infty }+\left(\mu_{0}-\mu_{\infty }\right){\left[1+{\left(\lambda \dot{\gamma }\right)}^{2}\right]}^{\frac{\left(n-1\right)}{2}}$$ (1)

where the infinite shear rate viscosity, $\mu_{\infty }$ , is 0.0035 Pa•s, the shear rate viscosity, $\mu_{0}$ is 0.056 Pa•s, model parameters λ and n are 3.313 and 0.368 respectively, and $\dot{\gamma }$ is shear rate (Cho and Kensey, 1991).

### No-slip condition

All vessel walls and the balloon occlusion were assumed to be rigid and no slip condition was prescribed. While this assumption can over-estimate instantaneous wall shear stress, the time-averaged wall shear stress is reliable and results in a less computationally expensive solution (Madhavan and Kemmerling, 2018).

### Numerical simulations

We solved the 3D incompressible, Non-Newtonian Navier-Stokes and continuity equations, shown in Eqs 2, 3 using finite element discretization.

$$\rho \frac{\partial u}{\partial t}+\rho \left(u∙\nabla \right)u=-\nabla p+\mu \nabla ∙\left(\nabla u+{\left(\nabla u\right)}^{T}\right)$$ (2)

∇∙u=0 (3)

where **u** is the fluid velocity vector, p is the hydrostatic pressure, ρ is the density of blood (1,060 kg/m^3^), and μ is the viscosity of blood (i.e., Carreau-Yasuda model, see Eq. 1).

The equations were solved by iterative, advanced, and fully coupled solvers. The mesh grid for each physiologic condition was refined to obtain mesh independence. In the present study, up to 2.5 million elements were used to discretize the geometry with a minimum element size of 0.09 mm and a maximum element size of 2.07 mm. Mesh adaptation studies were used to create finite-element meshes refined on regions with high pressure and velocity gradients to achieve convergence and less than 5% difference. The residuals for all simulations were less than 0.001 (i.e., <1E-3). This approach yielded stable, mesh independent and temporally independent results. Simulations were run until cycle-to-cycle periodicity was achieved in the pressure and flow fields. Typically, this resulted in performing simulations of three to four consecutive cardiac cycles. Simulations were set up and run on a desktop computer and the Wake Forest University (WFU) High Performance Computing Facility (Information Systems and Wake Forest University, 2021).

### Wall shear stress quantification

Changes in wall shear stress (WSS) and wall shear rate (WSR) are known to impact endothelial function and other physiological mechanisms within the vascular environment (Fu and Tarbell, 2013; Ebong et al., 2014). Therefore, we calculated time-averaged WSS (TAWSS) over a cardiac cycle at baseline, hemorrhage, and REBOA using Eq. 4:

$$TAWSS=\frac{1}{T}\overset{T}{\underset{0}{\int }}\left|\vec{WSS}\right|dt$$ (4)

Time-averaged wall shear rate (TAWSR) was calculated using Eq. 5:

$$TAWSR=\frac{1}{T}\overset{T}{\underset{0}{\int }}\left|\vec{WSR}\right|dt$$ (5)

To quantify the directional variations in WSS caused by the aortic occlusion during a cardiac cycle, the oscillatory shear index (OSI) was calculated using Eq. 6:

$$OSI=\frac{1}{2}\left(1-\left(\frac{|\overset{T}{\underset{0}{\int }}\vec{WSS}|dt}{\overset{T}{\underset{0}{\int }}\left|\vec{WSS}\right|dt}\right)\right)$$ (6)

## Results

**FIGURE 3:**
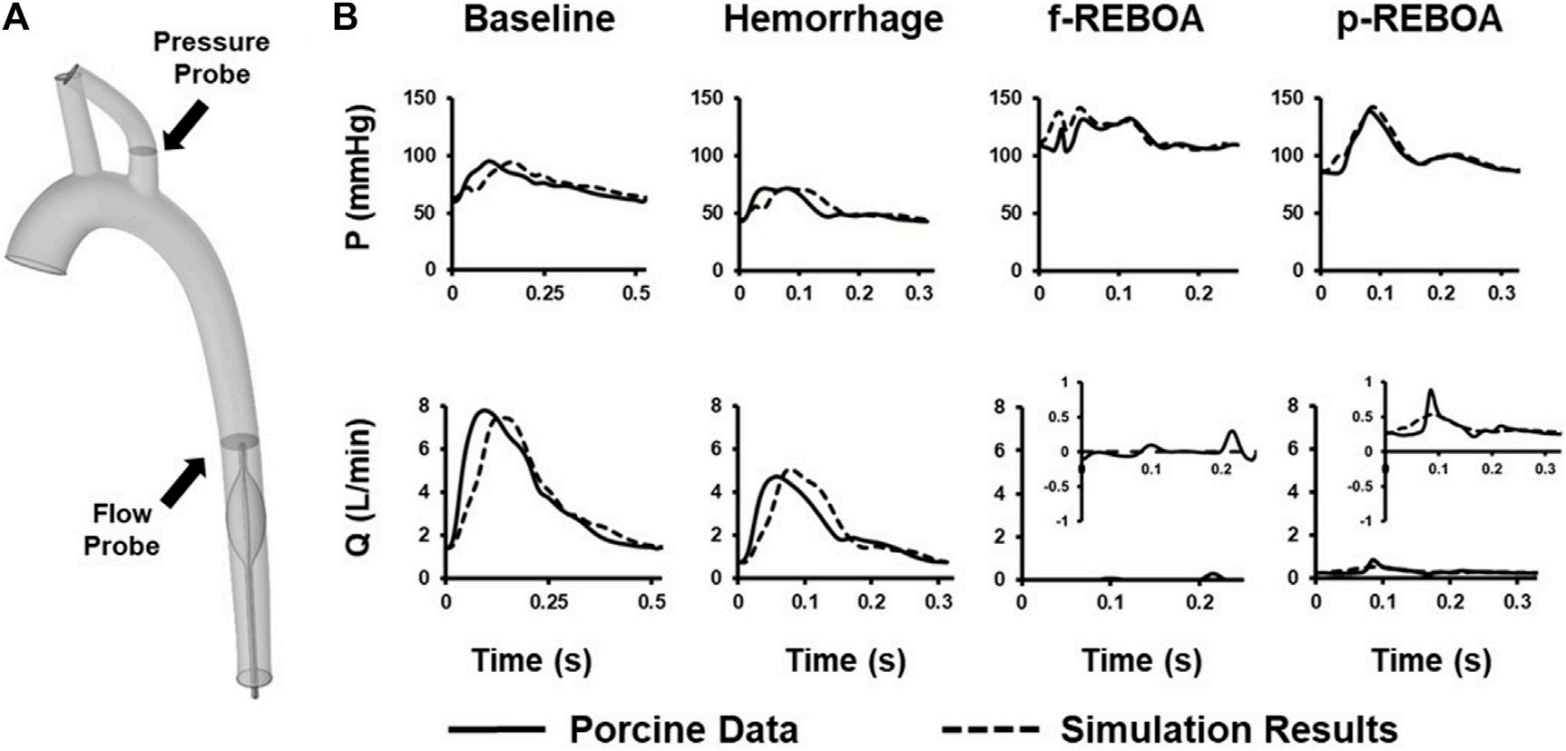
CFD Model Calibration. **(A)** Location of *in vivo* pressure and flow data used to calibrate CFD model on 3-D aortic geometry. **(B)** Comparison between measured pressure and flow data (solid line) and the simulation data (dashed line) calculated at the subclavian artery and thoracic aorta. A zoomed in representation of flow rate (Q) for both f- and p-REBOA is provided since recorded and simulated data were within a 1 L/min range. All simulated mean pressure and flow data are matched within a 5% error margin.

Hemodynamic simulations were performed at baseline (T0), end of hemorrhage (T30) and aortic occlusion (i.e., f-REBOA or p-REBOA) (T60) placement. To ensure congruent findings and minimize the bounds of error, numerical CFD simulation results were compared to *in vivo* experimental data of measured left subclavian arterial pressure and descending aortic flow waveforms. All simulation results of pressure and flow parameters were within 5% of averaged acquired hemodynamic data (Figure 3). The main difference between the rigid-wall simulation and experimental data was the time lag, approximately equal to 0.043s for the Baseline model, 0.015s for the Hemorrhage model and 0.005s for the p-REBOA model. This may be due to our relatively simplified imposed flow inlet waveforms. Nevertheless, our simulations provide good agreement with the experimental data.

**FIGURE 4:**
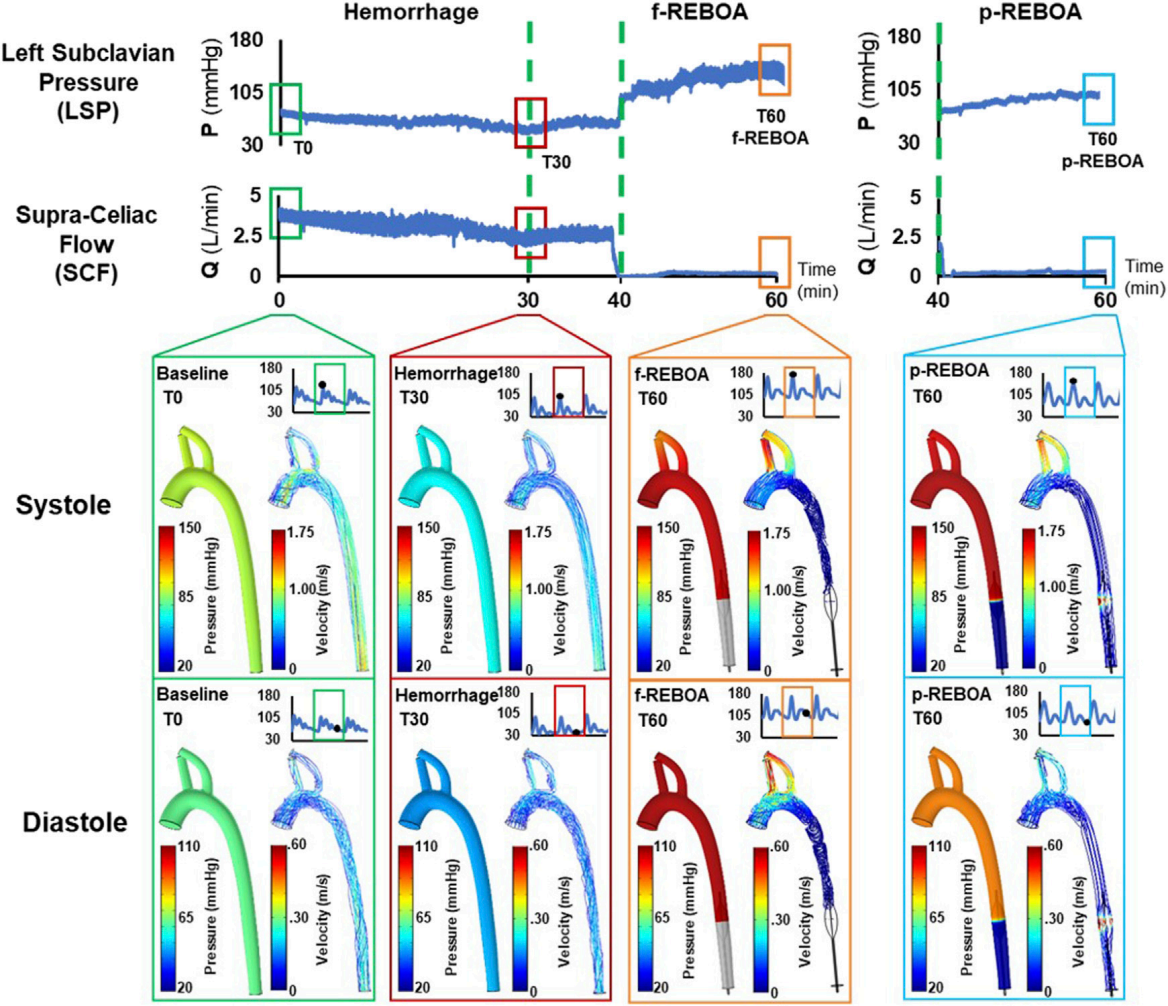
Pressure and velocity distributions. Results from the CFD simulations at systole and diastole are illustrated at baseline (T0), the end of 20% hemorrhage (T30), and post f-REBOA and p-REBOA implementation (T60).

**FIGURE 5:**
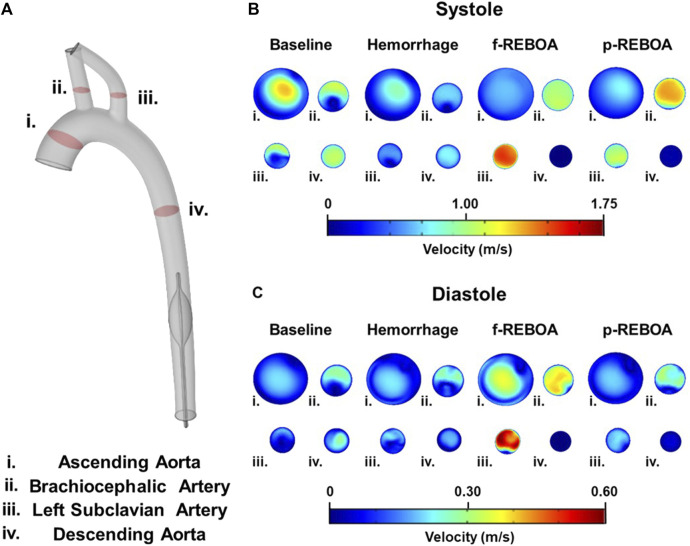
Axial flow profiles from the CFD simulations. **(A)** Illustration of cut planes used for flow visualization in the model. **(B)** Systolic axial velocity profiles are illustrated at baseline, end of 20% hemorrhage, and post f-REBOA and p-REBOA implementation. **(C)** Diastolic axial velocity profiles derived from the computational model are illustrated at baseline, end of 20% hemorrhage, and post f-REBOA and p-REBOA implementation.

Pressure and velocity distributions at systole and diastole for each phase (i.e., baseline, hemorrhage, f-REBOA, and p-REBOA) are illustrated in Figures 4, 5 and Supplementary Videos S1–S4. The Baseline model displayed systolic/diastolic blood pressure distributions consistent with normal porcine physiology (93/62 mmHg). As seen in Figure 4 and Supplementary Video S1A, maximum pressure is distributed at the distal side of the branching arteries within the supra-aortic arch (i.e., brachiocephalic and left subclavian artery). Flow in the ascending aorta was centralized but skewed marginally towards the outer wall in the aortic arch, as illustrated in Supplementary Video S1B. Blood flow accelerated in the upper arch and through the main supra-aortic branched arteries, while slowing down close to the inner arch. Due to the pressure gradient, a maximum velocity of 1.25 m/s was seen in this region.

Taking a deeper look into the secondary flows within the arch, we evaluated the velocity contours across four cross-sections in the 3D aortic geometry, illustrated in Figure 5A. Axial velocity contours at peak systole and diastole are illustrated in Figures 5B,C, respectively. During systole, velocity distribution in the ascending aorta was almost parabolic, but marginally skewed towards the inner wall with a maximum velocity of 1.21 m/s. Blood flow was more centralized throughout the descending aorta. Contrastingly, the velocity distribution in the brachiocephalic and left subclavian artery, and the proximal portion of the descending aorta skew towards the wall, with a max velocity of 1.00 m/s in the brachiocephalic artery and 0.95 m/s in the left subclavian artery. Qualitatively, the flow contours in the supra-aortic vessels form a C-shape (or epsilon-shaped), while the descending aorta displays more axisymmetric flow (Figure 5B). Here, the aortic curvature and curvature of branched vessels, induces secondary flows resulting in regions of separation and stagnation where axial velocity is less than 0.01 m/s. During diastole these characteristics are mostly consistent, however axial velocity in the ascending aorta skews towards the outer wall in a C shape, and flow in the descending aorta is distributed more towards the distal side of the aorta (Figure 5C). These flow patterns are consistent with previous reports of blood flow with the aorta (Vasava et al., 2012).

### Effects of hemorrhage on pressure and velocity distributions

The Hemorrhage model displayed a pressure of 74/42 mmHg which is a 42% reduction in comparison to the Baseline model, and is consistent with experimental data. As seen in Figure 4 and Supplementary Video S2, similar patterns of pressure and velocity spatial distribution were observed throughout the aorta, albeit at a lower magnitude to baseline conditions. During systole, a maximum velocity of 0.51 m/s was observed in the ascending aorta, which is nearly half the velocity observed in the Baseline model. Similarly, blood flow accelerates towards the supra-aortic vessels but skewed towards to the outer wall (Figures 5B,C). Past the aortic arch, velocity becomes increasingly more developed in the descending aorta. Axial velocity distribution patterns during both systole and diastole are similar to those present in the Baseline model.

### Effects of f-REBOA and p-REBOA on pressure and velocity distributions

Full and partial aortic occlusion (i.e., f-REBOA and p-REBOA) were successfully simulated following hemorrhage and can be viewed in Supplementary Videos S3, S4, respectively. Both f- and p-REBOA models demonstrate a rise in pressure and flow to the supra-aortic branches. Specifically, the f-REBOA model displays a proximal pressure of 147/103 mmHg which is 58% higher than baseline conditions (Figure 4). During systole, flow to the supra-aortic vessels (i.e., brachiocephalic and left subclavian arteries) increases by 196% compared to baseline and 303% increase compared to hemorrhage (2.10 m/s vs. 1.07 m/s and 0.69 m/s respectively). Qualitatively, axial velocity distribution in the ascending aorta displays a near parabolic profile but skewed towards the inner wall (Figure 5). Comparatively, the supra-aortic vessels experience a velocity distribution that is more similar to plug flow than seen in Baseline and Hemorrhage models. Lastly, evidence of flow disruption is most prominent in the descending aorta where the aortic occlusion imposes secondary flows and results in region of low flow and significant recirculation and flow stagnation. During diastole, f-REBOA exhibits a 195% increase in velocity compared to baseline and 247% compared to hemorrhage (0.74 m/s vs. 0.39 m/s and 0.30 m/s respectively). Secondary flow patterns past the aortic arch during diastole are qualitatively the same as in systole, simply lower in magnitude. However, in the ascending aorta axial velocity is near parabolic and skewed towards the outer wall in a D shape. Blood flow in the supra-aortic vessels display a C-shaped axial velocity distribution towards the outer wall (Figure 5C).

The partial aortic occlusion (i.e., p-REBOA model) displays pressure augmented to 144/86 mmHg which is 30% higher than baseline (Figure 4). During systole, average flow to the supra-aortic vessels is 174% higher compared to baseline and a 269% increase compared to hemorrhage (1.86 m/s vs. 1.07 m/s and 0.69 m/s respectively). Axial flow in the ascending aorta displays near parabolic flow skewed marginally to the inner wall. Velocity distribution in the supra-aortic vessels and regions of the descending aorta proximal to the occlusion are similar to f-REBOA. Contrastingly, p-REBOA results in flow acceleration around the balloon occlusion, increasing the near wall velocity distribution. Downstream of the occlusion flow is decentralized and skews towards the region of the wall where there was the most space between the balloon and wall (Figure 5B). During diastole, flow to the supra-aortic vessels displays a 91% increase in flow to supra-aortic vessels compared to baseline and 115% compared to hemorrhage (0.35 m/s vs. 0.39 m/s and 0.30 m/s respectively). The axial velocity distribution in the ascending aorta and supra-aortic vessels is qualitatively similar to those present in the f-REBOA, however to a reduced magnitude (Figure 5C).

Animations of the pressure and flow fields over an entire cardiac cycle, for baseline, end of hemorrhage, f-REBOA, and p-REBOA can be viewed in Supplementary Videos S1–S4.

### Effects of hemorrhage and REBOA on wall shear stress

At peak systole, the computed values of time averaged WSS (TAWSS) vary spatially, where the internal region of the ascending aorta is subject to larger WSS and the distal wall of the supra-aortic vessels, than the aortic arch and descending aorta. As expected TAWSS declines during hemorrhage, reflective of the reduced blood flow (43% decrease, 3.03 vs. 1.74 Pa).

**FIGURE 6:**
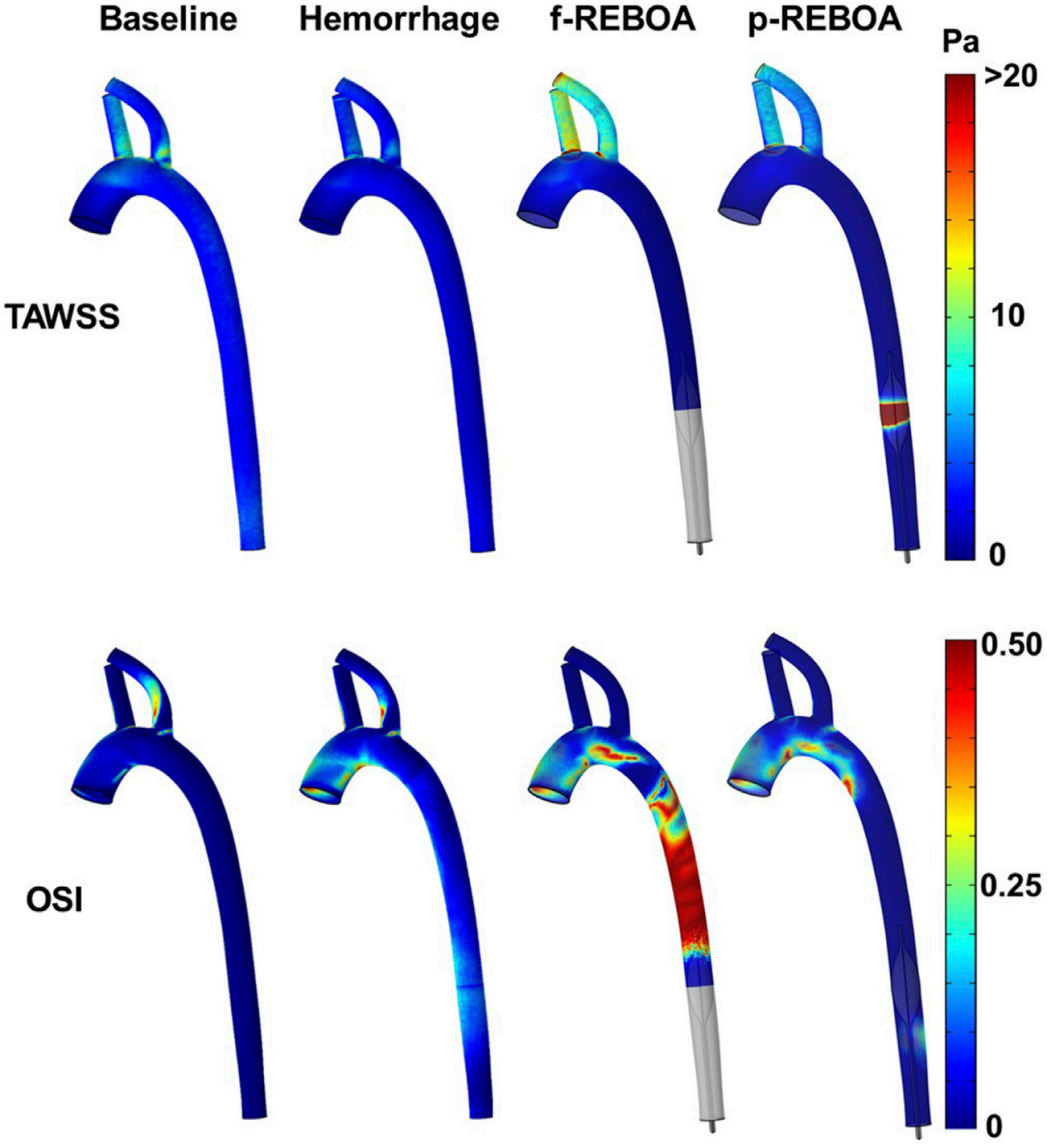
Shear stress and oscillatory shear index from the CFD simulations. Three-dimensional distribution of time averaged wall shear stress (TAWSS, top) and oscillatory shear index (OSI, bottom) over a cardiac cycle for baseline, hemorrhage, f-REBOA and p-REBOA are illustrated. Regions of high shear are observed around the p-REBOA. Significant flow oscillations are observed in the aorta during f-REBOA.

Both f-REBOA and p-REBOA models display significantly elevated WSS levels (>20 Pa) in the supra-aortic vessels, compared to both Baseline and Hemorrhage models. Here, TAWSS in the f-REBOA model is 97% higher than baseline values (9.04 vs. 4.58 Pa), while p-REBOA demonstrates a 22% increase (5.60 Pa). Contrastingly, the wall of the vascular geometry experiences different WSS trends around the site of occlusion as f-REBOA model displays near zero TAWSS levels while p-REBOA reaches 84.48 Pa. These results indicate that high degrees of partial aortic occlusion can significantly alter WSS, which may affect vascular endothelial and platelet function within this region (Figure 6).

Given the acute hemodynamic responses during hemorrhagic shock and aortic occlusion results in severe tachycardia, we also quantified TAWSR. TAWSR followed the same trends with the hemorrhage model demonstrating a 44.02% decrease across the aorta in comparison to the baseline model (443.9 1/s vs. 792.97 1/s). Additionally, the supra-aortic vessels in f-REBOA model experience a 2-fold increase compared to the Baseline model (2,464.85 1/s vs. 1,223.93 1/s) and the p-REBOA model displays a 22% increase in comparison to the Baseline model (1,501.50 1/s vs. 1,223.93 1/s).

Finally, due to the significant changes in flow patterns following the increase in heart rate upon 20% hemorrhage and subsequent aortic occlusion, we aimed to quantify the oscillatory shear index (OSI, Figure 6). The Baseline model displays an average OSI of 0.03 across the aortic geometry, while the Hemorrhage model displays an average of 0.09 which is 197% higher in comparison. Unsurprisingly, OSI values were highest proximal to the aortic occlusion, specifically in the f-REBOA model (0.31). f-REBOA resulted in a 137% increase in OSI in comparison to hemorrhage (0.21 vs. 0.09, respectively) and a 12-fold increase compared to the Baseline model (0.02). Similarly, p-REBOA displays a 306% increase in OSI proximal to the aortic occlusion compared to the Baseline model (0.07 vs. 0.02), however there is a 23% reduction when compared to the Hemorrhage model. Lastly, the f-REBOA experienced the highest OSI levels across all regions of the aortic geometry resulting in a 69.78% increase in average OSI in comparison to the p-REBOA model (0.19 vs. 0.06).

## Discussion

As hemorrhagic shock remains a leading cause of potentially preventable deaths in both civilian and military populations aged 18–45 years, REBOA has emerged as a promising technique to minimize internal bleeding (Eastridge et al., 2019). However, there is growing evidence that full aortic occlusion (i.e., f-REBOA) is not only challenging to implement but can also impose I/R injury, endothelial damage, acute coagulation changes, and has been associated with poor patient outcomes (Markov et al., 2013; Doucet and Coimbra, 2017; Thrailkill et al., 2021). As a result, partial aortic occlusion strategies have been proposed. However, there remains a significant gap in our knowledge and understanding of the acute hemodynamic and physiologic responses to REBOA. In this study, we integrated *in vivo* porcine measurements of pressure and flow into a CFD model simulating the acute hemodynamic responses during hemorrhagic shock and REBOA use. We successfully utilized the porcine model to 1) obtain physiologically relevant vascular geometries, 2) provide pressure and flow conditions, 3) calibrate outflow conditions, and 4) validate/confirm the simulation results. We observed significant changes not only in the magnitude of blood flow and pressure following aortic occlusion, but also changes in flow directionality and high shear rates.

At baseline, blood pressure and flow patterns throughout the porcine aorta were normal and consistent with previous reports of aortic blood flow (Vasava et al., 2012; Madhavan and Kemmerling, 2018; Shin et al., 2018; Deyranlou et al., 2020). Blood velocity profiles during hemorrhage were similar to the baseline model, albeit at a lower magnitude. This was expected since we observed a 20% reduction in CO during hemorrhage (6.0 L/min to 4.8 L/min) and corresponding drop in MAP from 74.74 to 54.44 mmHg in the animal model, similar to previously published studies (Gutierrez et al., 2004; Cannon, 2018). Given that we used the reduced inlet flow rates and pressure outlets for our CFD simulations of hemorrhage, our CFD results are similar to previous CFD models of hypotension and Class II hemorrhage (Vasava et al., 2012).

The inclusion of REBOA, in either full or partial aortic occlusion format resulted in an increase in blood pressure and velocity to the supra-aortic vessels (i.e., brachiocephalic and left subclavian arteries). Peak blood pressure was found in the upper aortic arch at a level of 146.8 mmHg in the f-REBOA model and 144.53 mmHg in the p-REBOA model. Similarly, velocity of blood increased to comparable flow rates observed at baseline. These results confirm that the REBOA device is able to augment and maintain perfusion to the cerebral circulation following hemorrhage (Stannard et al., 2011; Williams et al., 2018). However, they also suggest that perhaps a high degree of aortic occlusion may not be strictly necessary to augment pressure. Our CFD simulations with a high degree of partial occlusion (96.35% occlusion of diameter) reveal blood pressures in the range of 101 mmHg within the supra-aortic vessels, which is 86.96% higher than hemorrhage. Importantly, this estimate does not take into account the autoregulation of the carotid and cerebral vasculature, so there may indeed be a benefit for smaller partial occlusion or p-REBOA use (Russo et al., 2016c; Beyer et al., 2019).

On the flip side, it is important to note that REBOA serves two purposes: 1) to minimize internal bleeding within the chest or abdominal cavity, and 2) to augment blood pressure and perfusion to the heart and brain. By inserting the REBOA device, one induces an increase in proximal arterial blood pressure and systemic vascular resistance, that resembles aortic cross-clamping (Zammert et al., 2016; Beyer et al., 2019). Hence, higher degrees of occlusion may be initially preferred to rapidly control ongoing hemorrhage, initial clot formation, and provide maximal hemodynamic support. However, this may only be needed for short periods of time and would potentially be followed by transitioning to a partial flow state (Russo et al., 2016a; Williams et al., 2018). Future studies using this CFD platform can potentially aid in the optimization of balloon placement, timing, and weaning to offer maximum benefit. This will require further investigations that couple both acute hemodynamic and physiologic processes. While there is a well-established literature regarding the physiologic response to aortic cross clamping, there is a need to better understand the implications of REBOA on hemostasis, platelet function and hemostasis (Zammert et al., 2016; Khalid et al., 2022). Certainly, a lot of knowledge can be adapted from the aortic cross clamping literature.

In our simulation of f-REBOA, we observed considerable regions of low and stagnant flow proximal to the balloon. These findings match observations in preclinical animal models which report stagnant blood proximal to the balloon during full REBOA (Sadeghi et al., 2018). Regions of stagnated flow have been shown to be associated with thrombus formation (Hathcock, 2006; Diamond, 2016), and thus prolonged use of f-REBOA can potentially result in thromboembolic complications (Markov et al., 2013; Russo et al., 2016a). Similar to aortic cross clamping physiology, the lack of blood flow distal to the aortic occlusion elicits a metabolic acidotic response with increased lactate levels in ischemic regions (Falk et al., 1981; Russo et al., 2016c).

Partial aortic occlusion prevented blood stagnation by enabling flow across the balloon and to distal organs (Markov et al., 2013; Sadeghi et al., 2018). However, the high degree of partial occlusion (i.e., ∼90% occlusion by diameter) in our p-REBOA model displayed a region of high velocity and high shear around the balloon. These flow patterns are similar to other biomechanically driven investigations pertaining to the effect of vascular constrictions, such as aortic coarctations or stenoses in vascular regions such as intracranial, carotid, renal, and coronary arteries (Marshall et al., 2004; Yim et al., 2004; Wu et al., 2015; Tossas-Betancourt et al., 2020; Liu et al., 2021). We found the flow acceleration past REBOA consistent with previously reported findings on the effect of cardiovascular catheters in CFD simulations (Krams et al., 1999; Torii et al., 2007; Taebi et al., 2021).

The changes in TAWSS (>20 Pa) and TAWSR (>2,000 1/s) were most significant in the brachiocephalic and left subclavian arteries and the site of the partial aortic occlusion. These shear stress values were nearly 2 times higher than normal baseline conditions. Additionally, the descending aorta in the f-REBOA model displays elevated OSI levels proximal to the full aortic occlusion. While we recognize that the assumption of a rigid wall may result in the over-estimation of WSS, it is important to recognize that the acute changes in shear stress are not small. These elevated shear levels may have severe physiological consequences such as platelet activation, the disruption or shedding of the endothelial glycocalyx layer (Vasava et al., 2012; Ebong et al., 2014; Timmins et al., 2017), or at very high rates even denuding the endothelium lining. The shear-mediated shedding of the endothelial glycocalyx layer is of particular concern since it serves as the protective layer to endothelial cells and clinical studies have found that this barrier is highly susceptible to damage during hemorrhage alone (Rahbar et al., 2013; Johansson et al., 2017). The effect of partial or full REBOA on the endothelial glycocalyx layer is currently unknown but an interesting avenue of future study to more comprehensively understand the impact of this acute lifesaving intervention.

Given REBOA is currently meant to be a short-term lifesaving intervention, quantifying the impact of hemodynamic alterations to the pressure, flow and shear profile are of particular interest as they indicate potential areas of vascular vulnerability. This study aimed to better understand the hemodynamic changes and how they may contribute to the performance and implementation of REBOA clinically. We aimed to reach a higher level of integration by exploiting the hemodynamic data from our pig experiments for both the computational setup as well as internal validation/check of simulation accuracy.

However, this study is not without limitations. First, we only had access to experimental swine experiencing 20% total blood volume hemorrhage, which falls under Class II hemorrhage. While REBOA is commonly used for more severe hemorrhage conditions (i.e., Class III and IV, >30%), the current CFD model is representative of low MAP and high heart rates (i.e., tachycardia). Future studies can be done at these higher hemorrhage rates. Second, it is important to note that we did not have direct measurements of all cardiac flow waveforms in the pig experiments and thus made reasonable assumptions to create the flow inlet waveform and tuned the pressure outlet boundary conditions. While good agreement with experimental data was observed and computational simulations were within 5% of averaged *in vivo* hemodynamic data, further studies are needed to determine the sensitivity of the simulation results to changes in the inlet cardiac waveform and/or pressure outlet conditions. Third, we assumed rigid walls. Modeling wall compliance with spatial variations in material properties along the aorta can lend to more accurate numerical simulations for flow, pressure and most importantly wall shear stress. In general, a larger wall elasticity generally improves the agreement with experimental data. A study by Boccadifuoco et al. (2018), revealed by decreasing the elastic modulus (E) of the arterial wall, peak flow rate tends to become underestimated. Conversely as walls are stiffer or more rigid, peak flow rate tends to be over-estimated. A lower elastic modulus is also better for matching flow reversal regions within the aortic arch. Future work that takes a fluid-solid interaction approach can also integrate the vascular autoregulation that is experienced by the animal during hemorrhage.

Additionally, we did not model the actual blood volume loss during hemorrhage. Rather we took an open CFD approach where we simply modeled snapshots in time during each key experimental phase. Nevertheless, these simulations revealed important features of the acute hemodynamic response to hemorrhage and REBOA. This is an important limitation to note, since the results of our CFD models are a bit more generalizable to the response of full vs. partial aortic occlusion when the inlet flow conditions are low. Future studies will involve developing CFD models incorporating hemodynamic data from porcine hemorrhage models subjected to more severe levels of hemorrhage (i.e., 30%–50% total blood volume).

Future work can exploit the use of reduced order models to generate a closed loop model of the circulation and control for actual blood volume changes (Kim et al., 2010; Lau and Figueroa, 2015). We also acknowledge our limitation in modeling flow and oxygen transport past the aortic occlusion in this study. Herein, we focused on understanding the effects of a Zone 1 REBOA on flow and shear distributions within the ascending aorta and main supra-aortic vessels. However, in the future we plan to expand our CFD model to evaluate the impact of partial REBOA on the renal vasculature, oxygen delivery, and distal flow, as well as considerations for Zone 3 placement for pelvic injuries. With the availability of more robust *in vivo* animal data, future studies can investigate the effect of varying degrees of partial aortic occlusion in these alternative situations.

Finally, we acknowledge that the current CFD model is porcine-specific. One of the main reasons for developing this CFD model using pig anatomy and hemodynamic data was because we could achieve a higher quality of validation and control. As swine are an established preclinical model of hemorrhagic shock, the development of this CFD model allowed us to first establish the acute physiology and hemodynamics in a controlled manner. However, it is important to note that the methods in this study can be adapted to develop a human-based model in the future. This can be accomplished by taking human vascular geometry models and scaling pig hemodynamics to human-equivalent values. One of the major challenges with developing human specific CFD models for hemorrhage is the lack of robust hemodynamic data from hemorrhagic shock patients. In other words, while we could simulate a variety of scenarios, we would be limited in our ability to rigorously validate the numerical simulations. Nevertheless, the CFD models have translational value and can be highly informative for the design of new endovascular devices. It can also serve as a training tool for future medical professionals regarding endovascular hemorrhage control techniques.

## Conclusion

To our knowledge, this study is the first to develop and utilize CFD models to quantify the differences in acute hemodynamics during hemorrhage and intervention with REBOA. We identified significant changes in blood pressure and flow distributions during hemorrhage and REBOA. As expected, pressure and flow decreased during hemorrhage, reaching an average of 0.25 m/s in the descending aorta. Partial and full occlusion with REBOA augmented both pressure and flow to supra-aortic vessels to levels that were higher than baseline. This rise in blood velocity resulted in a significant elevation in WSS (>20 Pa). While the assumption of rigid walls could overestimate these WSS values, further studies are needed to determine optimal partial occlusion strategies that mitigate these regions of high shear, and poor perfusion during REBOA use. This study represents a critical step towards utilizing an *in silico* approach to quantify the local and systemic hemodynamics during hemorrhage and REBOA and/or other EHC implementation under the wide variety of conditions encountered clinically. Exploiting this CFD platform can potentially accelerate the optimization of REBOA and other EHC devices for improved design, implementation, and better patient outcomes following hemorrhage.

## Data Availability

The original contributions presented in the study are included in the article/Supplementary Material, further inquiries can be directed to the corresponding author.
