# Supplementary material for: Development of a computational fluid dynamic model to investigate the hemodynamic impact of REBOA
Source: Front Physiol. 2022 Oct 13;13:1005073. doi: 10.3389/fphys.2022.1005073 (PMC9606623; doi:10.3389/fphys.2022.1005073)
Supplement: Supplementary file 1 [file Presentation1.ZIP › Supplemental Videos_revised/Supplemental Video 2_Hemorrhage.pptx]

## Slide 1
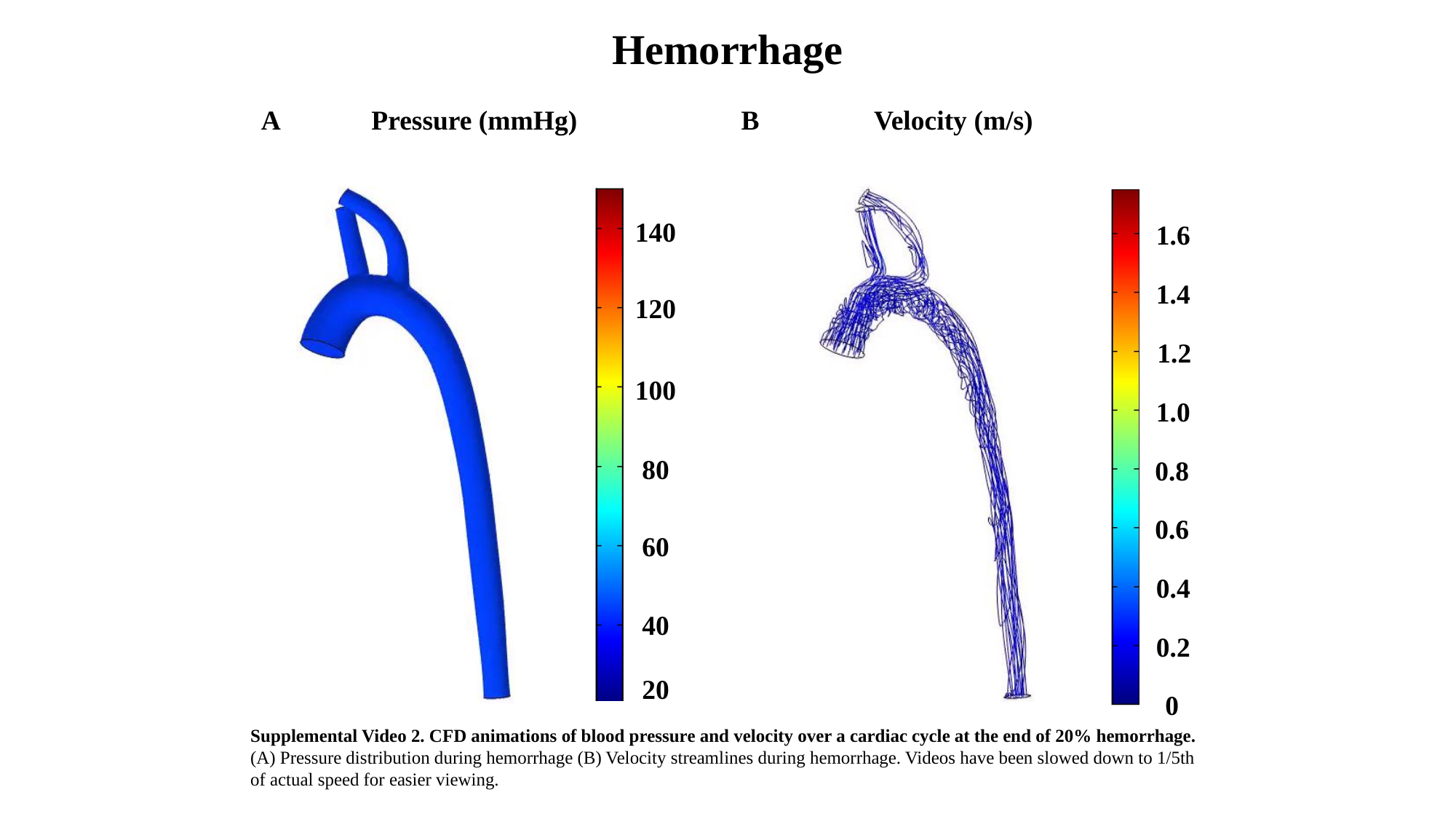

Hemorrhage
A
Pressure (mmHg)
B
Velocity (m/s)
140
1.6
1.4
120
1.2
100
1.0
80
0.8
0.6
60
0.4
40
0.2
20
0
Supplemental Video 2. CFD animations of blood pressure and velocity over a cardiac cycle at the end of 20% hemorrhage. (A) Pressure distribution during hemorrhage (B) Velocity streamlines during hemorrhage. Videos have been slowed down to 1/5th of actual speed for easier viewing.
