# Supplementary material for: Development of a computational fluid dynamic model to investigate the hemodynamic impact of REBOA
Source: Front Physiol. 2022 Oct 13;13:1005073. doi: 10.3389/fphys.2022.1005073 (PMC9606623; doi:10.3389/fphys.2022.1005073)
Supplement: Supplementary file 2 [file Image1.PDF]

Supplementary Material

Supplemental Video 1. CFD animations of blood pressure and velocity over a cardiac cycle during baseline. (A) Pressure distribution at baseline, (B) Velocity streamlines at baseline. Videos have been slowed down to 1/5th of actual speed for easier viewing.

Supplemental Video 2. CFD animations of blood pressure and velocity over a cardiac cycle at the end of 20% hemorrhage. (A) Pressure distribution during hemorrhage, (B) Velocity streamlines during hemorrhage. Videos have been slowed down to 1/5th of actual speed for easier viewing.

Supplemental Video 3. CFD animations of blood pressure and velocity over a cardiac cycle during full REBOA. (A) Pressure distribution during f-REBOA, (B) Velocity streamlines during f-REBOA. Videos have been slowed down to 1/5th of actual speed for easier viewing.

Supplemental Video 4. CFD animations of blood pressure and velocity over a cardiac cycle during partial REBOA. (A) Pressure distribution during p-REBOA, (B) Velocity streamlines during p-REBOA. Videos have been slowed down to 1/5th of actual speed for easier viewing.
